# COnsensus on Pediatric Pain in the Emergency Room: the COPPER project, issued by 17 Italian scientific societies

**DOI:** 10.1186/s13052-020-00858-9

**Published:** 2020-07-23

**Authors:** Franca Benini, Ilaria Corsini, Emanuele Castagno, Davide Silvagni, Annunziata Lucarelli, Luca Giacomelli, Angela Amigoni, Gina Ancora, Marinella Astuto, Fabio Borrometi, Rosa Maria Casilli, Elena Chiappini, Renato Cutrera, Arianna De Matteis, Giuseppe di Mauro, Anna Musolino, Andrea Fabbri, Federica Ferrero, Martina Fornaro, Michele Gangemi, Paola Lago, Francesco Macrì, Luca Manfredini, Luigi Memo, Annamaria Minicucci, Paolo Petralia, Nicola Pinelli, Roberto Antonucci, Silvia Tajè, Emiliano Tizi, Leo Venturelli, Stefania Zampogna, Antonio F. Urbino

**Affiliations:** 1 Paediatric Pain and Palliative Care Service, Department of Women’s and Children’s Health, University Hospital Padua, Via Giustiniani 3, 35126 Padua, Italy; 2 Department of Medical and Surgical Sciences, Unit of Paediatric Emergency, Sant’Orsola-Malpighi University Hospital, Bologna, Italy; 3 Department of Paediatric Emergency, Regina Margherita Children’s Hospital – A.O.U. Città della Salute e della Scienza di Torino, Turin, Italy; 4 Department of Paediatric Emergency and Critical Care, Azienda Ospedaliera Universitaria Integrata, Verona, Italy; 5 Department of Paediatrics and Emergency, Giovanni XXIII Children’s Hospital, A.O.U. Consorziale Policlinico – Giovanni XXIII, Bari, Italy; 6 Polistudium SRL, Milan, Italy and Department of Surgical Sciences and Integrated Diagnostics, University of Genoa, Genoa, Italy; 7 Accademia Medica Infermieristica di Emergenza e Terapia Intensiva Pediatrica (AMIETIP), Bologna, Italy; 8 Società Italiana di Neonatologia (SIN), Milan, Italy; 9 Società Italiana di Anestesia Analgesia Rianimazione e Terapia Intensiva (SIAARTI), Roma, Italy; 10 Società di Anestesia e Rianimazione Neonatale e Pediatrica Italiana (SARNePI), Roma, Italy; 11 Società Italiana di Pediatria Preventiva e Sociale (SIPPS), Roma, Italy; 12 Federazione Italiana delle Associazioni e Società Scientifiche dell’Area Pediatrica (FIARPED), Rome, Italy; 13 Osservatorio Nazionale Specializzandi Pediatria (ONSP), Rome, Italy; 14 Società Italiana di Medicina Emergenza Urgenza Pediatrica (SIMEUP), Milan, Italy; 15 Società Italiana di Medicina di Emergenza ed Urgenza (SIMEU), Turin, Italy; 16 Associazione Culturale Pediatri (ACP), Narbolia, Italy; 17 Federazione delle Società Medico-Scientifiche Italiane (FISM), Milan, Italy; 18 Associazione Italiana di Ematologia e Oncologia Pediatrica (AIEOP), Milan, Italy; 19 Società Italiana di Pediatria (SIP), Milan, Italy; 20 Associazione Ospedali Pediatrici Italiani (AOPI), Genova, Italy; 21 Federazione Italiana Aziende Sanitarie e Ospedaliere (FIASO), Rome, Italy; 22 Società Italiana di Pediatria Ospedaliera (SIPO), Milan, Italy; 23 Società Italiana Malattie Genetiche Pediatriche e Disabilità (SIMGePed), Milan, Italy

**Keywords:** Pediatric pain, Emergency department, Law 38/2010

## Abstract

In the pediatric setting, management of pain in the emergency department – and even in common care – is a challenging exercise, due to the complexity of the pediatric patient, poor specific training of many physicians, and scant resources.

A joint effort of several Italian societies involved in pediatrics or in pain management has led to the definition of the PIPER group and the COPPER project. By applying a modified Delphi method, the COPPER project resulted in the definition of 10 fundamental statements. These may represent the basis for improving the correct management of children pain in the emergency department.

## Introduction

Pain is frequently experienced in the pediatric age, both in primary care and in hospitals. Up 80% of hospital admissions are caused by diseases associated with pain, either acute or chronic [1–3]. This figure is even higher in the emergency department (ED) setting [4, 5]. Remarkably, pain is defined as a feared symptom; it is often present at the onset of a disease, it is always present during diagnostic and therapeutic procedures and it is associated with the fear of the disease itself [1]. Moreover, pain is a damaging symptom; it is widely accepted that alterations in neural activity due to pain and injury in early development may produce immediate and long-term effects on sensory processing and future response to pain [6]. Last, pain is associated with an increased anxiety and psychological discomfort; it also increases parental distress.

Therefore, the control of pain and stress in children is a vital component of emergency medical care [4]. In particular, timely administration of proper analgesic treatment can influence the outcomes of the medical procedure in the ED and can have a lasting effect on the perception of the child and his/her family about current and future medical care [1].

In Italy, Law 38/2010 is a precedent: the relief of pain is a child’s “right to health”. It is an ethical mandate to appropriately treat pain and suffering in pediatric patients [7]. However, pain is sometimes poorly managed in the ED [4, 8]. Barriers to the assessment and treatment of pain in children include a lack of knowledge regarding pain and its consequences on brain development, pain treatment, unfamiliarity with new products and techniques, fear of the adverse effects of the medication, personal values and beliefs, and lack of resources. Proper assessment of pain also has potential medical–legal implications.

Therefore, a joint effort of 17 Italian Scientific Societies (see acknowledgments) who are active in the field of pain management and pediatrics established the PIPER group (Pain in Paediatric Emergency Room), with the aim to reach a COnsensus on Paediatric Pain in the Emergency Room (COPPER). Here, we summarize the key findings of the COPPER project.

## Methods

The COPPER project was independently established by the PIPER group in September 2018. A Study Group was established within the members of PIPER to review the literature and draft a consensus document and some statements regarding the management of pain in children admitted to the ED.

The PIPER group focused on five main fields: ethics and deontology, prevalence, pathogenesis, evaluation, measurement, and acute and procedural pain therapy. For each field, international and Italian national guidelines, metanalysis, systematic reviews, consensus documents and position papers published on PubMed, EMBASE and Cochrane Library in the last 10 years were reviewed; the Italian Health Department recommendations were also considered. The most relevant papers were selected for drafting the consensus document.

A total of 20 statements were prepared by the Study Group and evaluated by the Panel of Experts in December 2018. In this phase, the statements were improved, and the redundant parts were excluded or merged. The statement developed for the ethics, law and deontology field was considered to be a fundamental premise for the entire document and was excluded from the evaluation of consent since it related to well-defined national and international obligations.

In January 2019, a consensus meeting was held, and each statement was discussed, modified and evaluated collectively using a modified Delphi method, in line with similar efforts [9], which led to the approval of 11 statements. The approved statements were then corrected by the Study Group and reduced to 10: 1 on prevalence, 1 on pathogenesis, 2 on evaluation and measurement, 5 on therapy and 1 dedicated specifically to pain management in children with neurocognitive problems.

The 10 statements were then assessed by the Panel of Experts again using the modified Delphi method in April 2019. The definitive consensus document was submitted to the members of each Scientific Society and Association and was finally approved in May 2019.

## Results

The COPPER panel agreed that access to pain care is a fundamental human right. Preventing and appropriately treating pain is an ethical, deontological and legal obligation to which all health professionals are bound, and which is prescribed by Law 38/2010 of the Italian Republic, the Code of Ethics of Nurses and in the Medical Code of Ethics. The 10 statements are reported in Table 1.

**Table 1:** Management of pediatric pain in the ED and beyond: the 10 statements of the COPPER project

1. Pain is a frequent, fearful and harmful symptom in newborns, children and adolescents in all clinical conditions; it undermines health and is a source of anxiety and worry.	
2. All children perceive pain; the younger the child, the higher the level of his/her perception and the consequent damage.	
3. Pain must always be assessed and measured in children. Pain measurement must be carried out by validated tools adapted to age and setting; the data reported must be recorded on a medical chart.	
4. Pain measurement must be always carried out at the first contact with the patient during medical examination, before discharge from the ED, whenever a child seems to be in pain or complains about pain, whenever the caregivers report the child being in pain and also in order to assess the efficacy of the planned analgesic treatment.	
5. Pain must be always promptly treated using both nonpharmacological and pharmacological procedures.	
6. The presence of caregivers in close contact with children is the mainstay of pain management and must be assured. The analgesic treatment recommended must always be communicated and shared with the child and/or with the relatives and caregivers using appropriate and effective communication tools.	
7. All analgesic drugs must be chosen considering the type and intensity of the pain; they must be appropriately prescribed according to age, weight and clinical situation. The route of administration must be as noninvasive as possible and must be the most effective. In the event of insufficiently controlled pain, a rescue dose must be established.	
8. Procedural pain must be always predicted. Any unnecessary procedure must be avoided.	
9. When discharging a child treated for pain, caregivers should receive correct information regarding the management of potential recurrence and the timing of the medical check-up with general pediatrician/practitioner.	
10. Pain in children with motor and/or cognitive impairment can be difficult to recognize. In these patients, pain must always be assessed and measured with specifically validated tools and must be treated according to an analgesic plan that considers the global situation of these patients, peculiarity of the causes of the pain and, if provided, of the current medication being taken.	

## Conclusions

In the pediatric setting, management of pain in the ED – and even in common care – is a challenging exercise, due to the complexity of the pediatric patient, poor specific training of many physicians, and scant resources [1]. A joint effort of several Italian Societies involved in pediatrics or in pain management has led to the definition of the PIPER group and the COPPER project. The COPPER project resulted in the definition of 10 fundamental statements, which will represent the basis for an actual real change in the correct management of children pain in the ED.

## Data Availability

Not applicable.

## Abbreviations

COPPER: Consensus on Paediatric Pain in the Emergency Room
ED: Emergency department
PIPER: Pain in Paediatric Emergency Room
